# The Feasibility Study of a Revised Standard Care Procedure on the Capacity of Nasogastric Tube Placement Verification Among Critical Care Nurses

**DOI:** 10.1097/jnr.0000000000000302

**Published:** 2019-07-16

**Authors:** Feng-Huang Yang, Feng-Yu Lin, Yueh-Juen Hwu

**Affiliations:** 1 MSN, RN, Deputy Director, Department of Nursing at Chung Kang Branch, Cheng Ching Hospital; 2 PhD, Associate Professor, General Education Center, Overseas Chinese University; 3 PhD, RN, Professor, College of Nursing, Central Taiwan University of Science and Technology; 4 Contributed equally.

**Keywords:** critical care, intervention study, nasogastric tube, placement verification

## Abstract

**Background:**

Evidence-based studies propose that the aspirate pH test may be easily and reliably conducted to verify the proper placement of nasogastric tubes (NGTs). Nurses rarely implement this procedure because of the lack of related knowledge.

**Purpose:**

The purpose of this study was to explore the feasibility of implementing a revised standard care procedure to enhance nurses' ability to verify placement of the NGT.

**Methods:**

his study used a quasi-experimental, longitudinal research design. Nurses from two intensive care units were randomly assigned to the experimental group (*n* = 35) and the control group (*n* = 31). A revised standard-of-care procedure to confirm the proper placement of an NGT was incorporated into a slideshow presentation, a printed leaflet, and an audit checklist. The experimental group received continuous education and individual teaching on the revised standard-of-care procedure, whereas the control group did not receive additional education and continued to provide conventional care. The study gathered data using scales designed to address knowledge of and attitudes toward verification of NGT placement and the checklist for auditing the NGT care procedure. Scales were implemented before and after the practice program was conducted, in Months 1, 2, and 3, to evaluate the feasibility of the developed improvement measures.

**Results:**

This study found significant improvements in the experimental group in terms of knowledge regarding NGT placement verification and the NGT care auditing procedure. The positive improvement of the intervention on the NGT care auditing procedure remained for at least 3 months after the end of the intervention.

**Conclusions:**

The findings suggest that using an aspirate pH test is a feasible approach to verify NGT placement in critical care units, a crucial aspect of care necessary to promote patient safety and quality of care.

## Introduction

Patients in intensive care units (ICUs) often experience inability to eat orally and surgery-related dysphagia, improper placement of the endotracheal tube, stroke, and other issues. Some researchers have found that critically ill patients experience delays in enteral feeding initiation and frequently miss meeting nutrition targets (Stewart, Biddle, & Thomas, 2017). Malnutrition, an important issue in the care of the critically ill, is associated with increased costs of care and poor patient outcomes. Inserting a nasogastric tube (NGT) is a measure frequently used to resolve these problems.

Patients of any age may require NGT placement. Thus, the safety of this procedure is worth discussing. NGT placement may increase the risk of resistance and struggle by patients and result in a greater probability of using physical restraints and of unplanned extubations (Lin, Liao, Yu, Chu, & Ho, 2018). The improper placement of NGT may threaten the safety of patients, especially critically ill patients in ICUs (Bourgault et al., 2014). According to a recent literature review (DiBardino & Wunderink, 2015), aspiration pneumonia should be a consideration for critical care patients who are on NGT feeds. Incorrect NGT placement and aspiration place patients at risk. Therefore, NGT placement verification is of great importance in the proper care of ICU patients.

At present, no single, nonradioactive method exists for verifying NGT placement. The current gold standard for NGT placement verification is X-ray. Evidence from numerous research studies has found that the air bolus method and the resultant aspirates are unreliable and cannot correctly determine the placement of NGT. Using multivariate methods to verify NGT placement is thus preferable (Chan et al., 2012; Jiang, Lin, Kao, Lin, & Wu, 2013). Simple and inexpensive detection methods have also been considered clinically, and the aspirate pH test was determined to be the most reliable and economical method for bedside verification of NGT (American Association of Critical-Care Nurses [AACN], 2016).

According to the evidence-based literature, X-rays and aspirate pH tests are most frequently used in the ICU to verify placement of an NGT. In a 3-month study of 100 ICU patients with NGT, all nurses (*N* = 42, 100%) used the aspirate pH test. Only 10 patients received X-ray to verify NGT placement, indicating that it is not feasible even in the ICU (Ke, Lin, Hsieh, Hwu, & Chang, 2014).

To ascertain nurses' knowledge about methods for NGT placement verification and behaviors, this study used a structured questionnaire to survey 200 nurses with direct patient care responsibilities at one regional hospital. One hundred ninety-five valid questionnaires were received and used in subsequent analysis work (response rate: 97.5%). The results revealed that more than half of the participants (50.3%–65.6%) could not answer questions related to the aspirate pH test. Only 4.6% of the participants had used the aspirate pH test to verify NGT placement (Yang, Lin, & Hwu, 2017). This result indicates that many nurses are unfamiliar with the aspirate pH test to confirm NGT placement.

Verifying the correct placement of NGT in critical care settings is imperative and frequently the sole responsibility of nurses. Methods currently in use include obtaining the aspirates (45.6%) and auscultation with insufflation of air (41.5%; Yang et al., 2017); therefore, an additional aspirate pH test to confirm NGT placement is feasible. The incidence of NGT misplacement can easily be significantly reduced when nurses follow revised standard care procedures to confirm NGT placement (Eveleigh, Law, Pullyblank, & Bennett, 2011). Thus, the aim of this quasi-experimental study was to investigate whether a revised standard care procedure could significantly improve NGT placement verification among critical care nurses.

## Methods

### Study Design and Participants

This study used a quasi-experimental, longitudinal research design and was conducted in two medical–surgical ICUs at one regional teaching hospital in central Taiwan. These two units were similar in terms of the number of beds and personnel. Cluster randomization was used to assign these units as either the experimental group or the control group to avoid cross-contamination. This study was approved by the ethics committee of the participating hospital (HP160043). G-Power Version 3.1.9.2. (Heinrich Heine Universitat, Dusseldorf, Germany; Faul, Erdfelder, Lang, & Buchner, 2007) was used to calculate the sample size. As no prior study had addressed the specific issue taken up in this article, a medium effect size of .5, a significance value (α) of .05, and a statistical power (1 − β) of .95 were used (Cohen, 1992). On the basis of these measurements, a minimum sample size of 54 participants was determined. The inclusion criteria were nurses who had worked in the ICUs for more than 3 months, had completed the consent form, and were willing to participate in the study. All of the nurses in the two units met the inclusion criteria and agreed to join in this intervention study. Thirty-five nurses were in the experimental group, and 31 were in the control group.

### Intervention

A four-step theoretical domains framework was used to develop the intervention (French et al., 2012). Step 1 identified target behaviors and capabilities related to NGT placement verification. Step 2 chose the theoretical framework most likely to elicit the process of learning effects. Step 3 designed the contents of the NGT practice program. These three steps helped preserve the intellectual integrity of NGT placement verification capabilities. Step 4 used subjective (structured questionnaire to determine nurses' knowledge and attitudes toward the NGT placement verification method) and objective (the checklist for auditing the NGT care procedure) outcomes to evaluate the capacity of NGT placement verification among participants. In addition to relevant knowledge, nurses require practical competence in NGT placement verification (i.e., “know-what” vs. “know-how” knowledge; Schunk, 2007). To gather these data, researchers designed the intervention as follows.

A revised NGT placement verification step was developed based on the literature (AACN, 2016; Metheny & Titler, 2010; Stepter, 2012; Tan, Chang, & Wu, 2012), a quality improvement project (Ke et al., 2014), and the results of a survey (Yang et al., 2017) and was added to the standard care procedure. This intervention was named “You must know the revised standard of care procedure for confirming placement of NGT.” The contents of this intervention are described below.

1. Reason for procedural change
  (1)Evidence from many research studies shows that the aspirate obtained and the air bolus methods are unreliable and cannot correctly determine the gastric placement of NGT.
  (2)Although radiographic imaging is the present gold standard for NGT placement verification, it is not feasible for use in critical care settings.
  (3)The most reliable and economical method for verification of NGT at the bedside is the aspirate pH test.
2. Practice recommendations
  (1)Current bedside practice in Taiwan: (a) Aspirate obtained is the primary method, followed by the auscultation of air bolus method. (b) Radiographic imaging is used occasionally.
  (2)AACN recommends that the most reliable and economical method for verifying NGT at the bedside is the aspirate pH test.
3. Key elements of the revised standard care procedure
  (1)An initial X-ray is recommended if unable to confirm NGT placement before administration of a substance via the NGT.
  (2)If no substance will be introduced into the NGT (suction or clamped), verify placement via absence of respiratory symptoms and aspiration of gastric contents.
  (3)Subsequent verifications of tube placement must be done before each feeding and administration.
  (4)Obtain aspirate 0.5–1 ml to check the color.
  (5)Test aspirate on pH indicator paper (pH between 1 and 5.5).
  (6)Combine one of the other methods as follows:
    ˙Check whether the tube twines in the mouth.
    ˙Check tube marking and/or tube length.
  (7)Check if the patient is on acid-inhibiting medication.
  (8)Nurses must document placement of the NGT every 4 hours and before the administration of any substance.

The amended NGT placement verification procedure was organized as a slideshow presentation, printed on leaflets, and used to develop an audit checklist to promote nurse awareness and application. In addition, two sessions of in-service education were arranged. Written information was distributed to all of the nurses, who were required to read the contents carefully, apply the procedure in a care situation, and propose amendment suggestions in the morning meeting 1 week later. When nurses conduct the aspirate pH test, they may encounter problems such as no aspirate and the need to interpret color change (Boeykens, Steeman, & Duysburgh, 2014). Thus, the head nurse provided instruction at the bedside using the audit checklist for the NGT care procedure to (a) understand the actual problems and difficulties encountered by nurses during implementation of the procedure and (b) provide assistance intended to increase the consistency and correctness of implementation. The NGT placement verification flowchart was posted on the wall in the ICU to remind nurses to verify NGT placement before administration or feeding and to check whether the patient was on acid-inhibiting medication (e.g., H_2_ receptor antagonists, proton pump inhibitors; Fan, Tan, & Ang, 2017).

The revised standard care procedure for NGT placement verification was implemented for 2 weeks. After the third posttest, the revised standard care procedure of NGT placement verification continuing education was held for the nurse participants in the control group. These interventions reflect the best learning practices for the clinical setting, which should incorporate reminder, audit, and feedback procedures (French et al., 2012).

### Measures

#### Questionnaires addressing the knowledge and attitudes of nurses toward the nasogastric tube placement verification method

Structured questionnaires were used to ascertain the knowledge and attitudes of the participants toward NGT placement verification. The demographic data collected included age, gender, credentials, years of nursing experience, and the ratio of patients fed via NGTs every day.

Ten items on the questionnaire addressed the knowledge of participants regarding NGT placement verification. Examples included identification of both the best method of NGT placement verification and NGT dislocation. Each correct answer earned a score of 1, and each wrong answer earned a score of 0; the total possible score range was 0–10, with higher scores associated with better knowledge of NGT placement verification. Four items on the questionnaire addressed respondent attitudes, with the aim of discerning opinions on the revised standard care procedure. Scores were based on a 5-point Likert-type scale, with 1 = *strong disagreement* and 5 = *strong agreement*. The total possible score range for this section was 4–20, with higher scores associated with a more positive attitude toward using the revised standard care procedure.

Content validity was verified by a panel of four experts (nursing professor, nursing supervisor, nursing practitioner, and physician). Each item was scored on a scale of 1–5, with 5 indicating highest appropriateness and applicability. Items with a mean panel-wide score of less than 4 were deleted, resulting in a final questionnaire of nine items. The final questionnaire earned a content validity index of .90. Reliability testing was conducted after the questionnaires were collected from the participants. The knowledge-related items, scored dichotomously as either right or wrong, earned a Kuder–Richardson coefficient of .88. The attitude-related items, scored based on a 1- to 5-point scale, showed an internal consistency Cronbach's alpha of .86.

#### Checklist for auditing the nasogastric tube care procedure

The checklist for auditing the NGT care procedure was developed to evaluate and monitor the integrity of the participants' implementation of the NGT care procedure. The NGT care procedure was divided into six major criteria: (a) implementation of cleaning skills required by the procedure, (b) arranging the patient position during and after administration and feeding, (c) verifying NGT placement, (d) feeding or administering drugs after NGT placement verification, (e) maintaining NGT patency, and (f) recording the observations and assessing and managing patients. Each criterion had its own items (subitems), with 17 subitems in all. The 17 subitems were categorized into “achievement,” “failure,” and “not applicable.” The full score was 17 points, no points were deducted for “not applicable” answers, and 1 point was deducted for the failure of each subitem to evaluate the nurses' NGT care behavior. A pilot study of the checklist for auditing the NGT care procedure was conducted in the ICU of another regional teaching hospital (Wu, Lin, Hwu, Ke, & Chang, 2016). “Nurse informs physician if unable to confirm placement and consider X-ray” was added to this study, resulting in a final audit checklist of 18 items.

The two measurement tools previously discussed were used to evaluate the ability of the participants to verify NGT placement both before and at 1, 2, and 3 months after the intervention. The pretest and posttests were conducted by a nurse who was responsible for quality assurance to maintain the consistency of the evaluation.

### Data Collection

**Figure 1.**
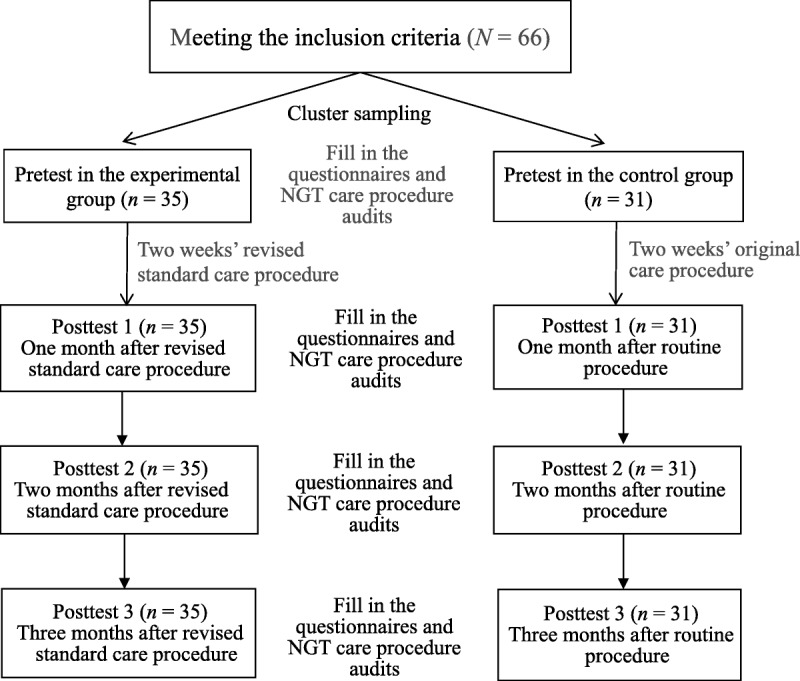
Study flow diagram. NGT = nasogastric tube.

Data were collected from November 2016 to May 2017. Before the intervention of the revised standard care procedure, the participants in the experimental and control groups completed the “Structured Questionnaire of Nurses' Knowledge and Attitudes Regarding the NGT Placement Verification Method” and “Checklist for Auditing the NGT Care Procedure.” The nurses in the experimental group then received 2 weeks of training on the revised standard of care procedure for confirming placement of NGT, whereas nurses in the control group implemented NGT care procedures according to conventional practice. To explore the sustainable effects of behavioral change, nurses in both groups were given a posttest evaluation of knowledge and attitudes toward NGT placement verification and the checklist for auditing the NGT care procedure at 1, 2, and 3 months after the intervention to compare the immediate and longer-term effects of the intervention (Figure 1).

### Data Analysis

Data were analyzed using IBM SPSS Statistics Version 23.0 (IBM, Armonk, NY, USA). Demographic and outcome characteristics were analyzed using descriptive statistics. A chi-square test and independent *t* tests were used to verify homogeneity between the groups at baseline. To assess the interpretability of the main effects of the intervention between the groups, a separate analysis of covariance (ANCOVA) was conducted by adjusting for baseline on the outcome measures. Finally, to ensure the learning effects of the revised standard care procedure over time, the knowledge and attitudes of participants toward NGT placement verification and NGT care procedure audit responses were compared among the four time points (pretest and three posttests). Between-group differences in the outcomes were analyzed using general linear modeling analysis and a repeated-measures ANCOVA (RANCOVA).

## Results

### Sample Characteristics

Figure 1 presents the study flowchart. General characteristics such as gender, age, education, years of nursing experience, and ratio of patients requiring NGT feeding showed no significant differences between the two groups, which supported intergroup homogeneity. Moreover, with regard to the three pretest numerical values of the nurses in both groups, although no significant difference in knowledge was found, the results of the attitude and care procedure audit in the control group were superior to those of the experimental group (Table 1).

**TABLE 1.**
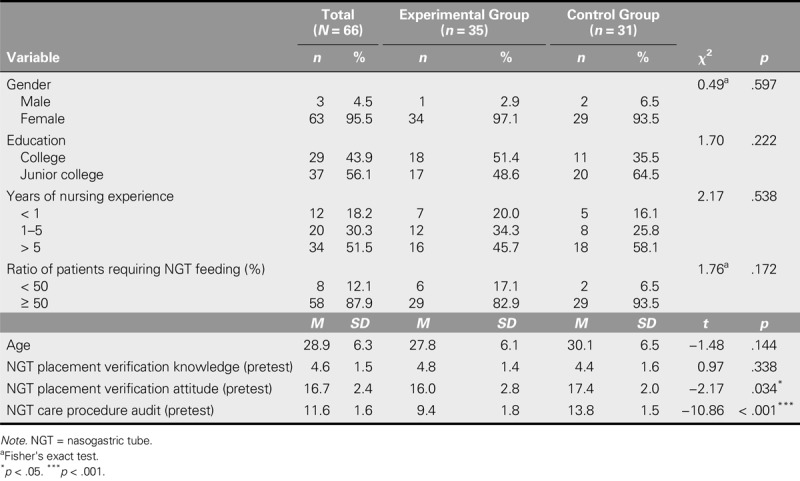
Demographic and Outcome Variables of Participants at Baseline (*N* = 66)

### The Immediate and Longer-Term Effects of the Revised Standard Care Procedure on Nasogastric Tube Placement Verification Ability

After using ANCOVA to eliminate the interference of the pretest between both groups (Tu, 2017), the posttest results showed that the experimental group had significantly greater knowledge of NGT placement verification and auditing of NGT care procedure than the control group.

General linear modeling and a sphericity test were used to analyze correlations between changes in outcome variables (knowledge, attitude, and audit levels) over time. RANCOVA with least significance difference was used to examine differences in outcome variables across the four time points.

### Knowledge of Nasogastric Tube Placement Verification

**Figure 2.**
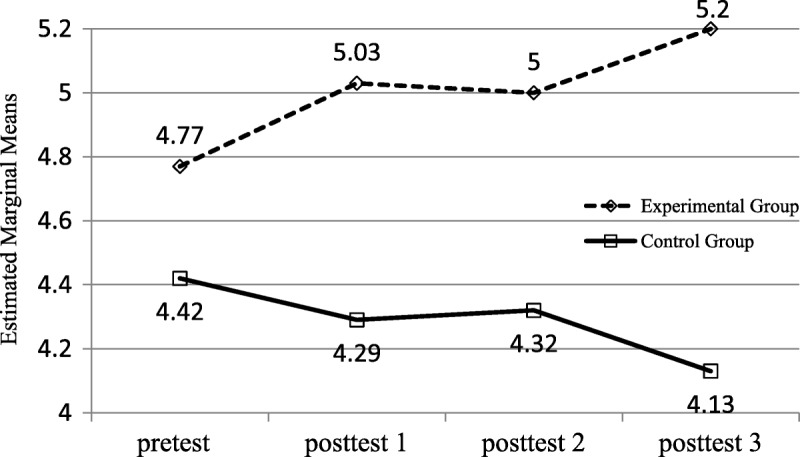
Mean scores for knowledge of NGT placement verification over time.

The assumption of sphericity was met (*p* > .05). It meant that there were no significant correlations among the four repeated measures. The results from RANCOVA revealed a significant group effect on knowledge of NGT placement verification, *F*(1, 33.12) = 6.93, *p* = .011, with a higher postintervention mean difference in the experimental group relative to the control group. In terms of time effect, the change in knowledge of NGT placement verification over time was not significant in either group (Table 2). In addition, there was no significant Group × Time interaction effect on knowledge of NGT placement verification between the groups over time (*p* = .496), as the degree of increased knowledge of NGT placement verification for both groups tended to converge over time (Figure 2).

**TABLE 2.**
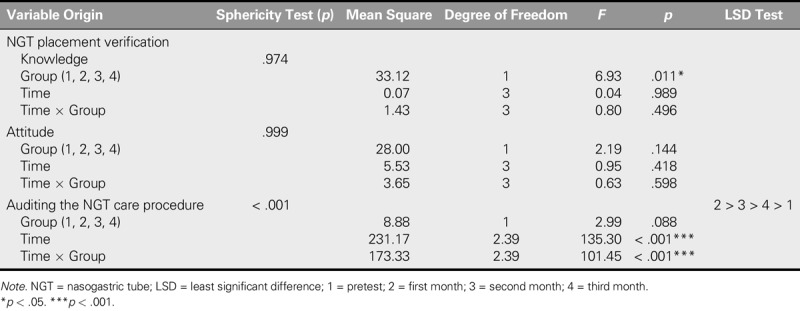
Lasting Effects in Capacity of NGT Placement Verification

### Attitudes Toward Nasogastric Tube Placement Verification

RANCOVA found no significant main effects of group, time, and Group × Time interaction on attitude. The attitudes toward NGT placement verification in the experimental group rose after the first month and then fell, which was still lower than the pretest after the third month. However, the attitudes in the control group continued to fall until stabilizing after the third month.

### Auditing the Nasogastric Tube Care Procedure

**Figure 3.**
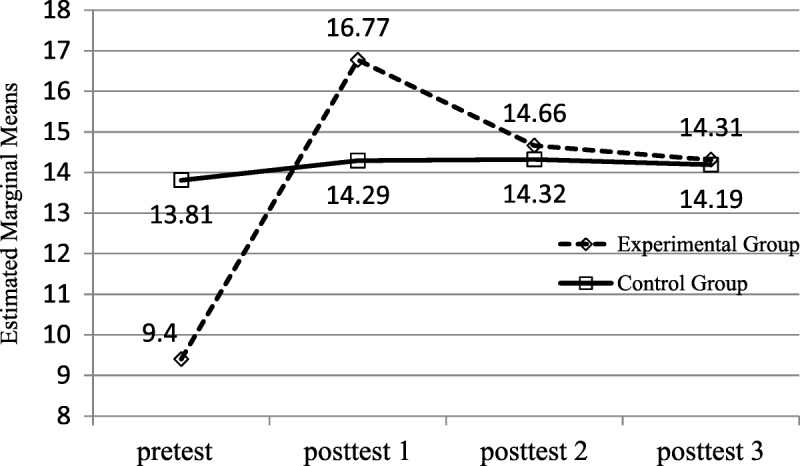
Mean scores for auditing of NGT care procedure.

The assumption of sphericity was not supported (*p* < .05). A Greenhouse–Geisser correction was conducted because of the significant correlations among the four repeated measures. After a significant repeated-measures result, pairwise comparisons with the least significance difference were used to determine at which points the auditing of NGT care procedure differed. RANCOVA adjusted the baseline of the NGT care procedure audit to examine the time effect of the changes in score between groups but did not confirm the significant group effect, *F*(1, 8.88) = 2.99, *p* = .088. In terms of time effect, RANCOVA revealed a statistically significant improvement in the NGT care procedure audit, *F*(2.39, 231.17) = 135.30, *p* < .001. This means that the audit score changed over time. There was also a significant Group × Time interaction effect on the NGT care procedure audit between groups over time, *F*(2.39, 173.33) = 101.45, *p* < .001 (Table 2; Figure 3). Hence, there was an intergroup difference in time effect.

Pairwise comparisons revealed that the pretest for the NGT care procedure audit yielded significantly different scores from those obtained in the 1-, 2-, and 3-month posttests. The frequency of NGT care procedure audits in the experimental group was greater than that in the control group, and differences between the four scores all achieved significance. This indicated that the intervention of the revised standard procedure achieved a lasting increase in NGT care procedure audits in the experimental group.

## Discussion

This article is the first quasi-experimental study to assess the effectiveness of a revised standard care procedure on NGT placement verification in a sample of critical care nurses. The study results show that the scores of NGT placement verification knowledge and NGT care procedure audit in the experimental group were higher than those in the control group. The intergroup differences in NGT care procedure audits persisted across all posttest time points. However, the time effects were not seen in the knowledge of NGT placement verification. The auditing score in the experimental group, lower than that in the control group at pretest (Table 1), significantly improved after the intervention. The main issue addressed in this article was whether the time effects of score changes between the two groups supported significant group effects. Therefore, posttests were carried out at 1, 2, and 3 months after completion of the 2-week intervention. Table 2 shows that knowledge and attitudes toward NGT placement verification did not exhibit longitudinal effects and that the NGT care procedure audit did exhibit significant longitudinal effects. This finding suggests that a period of reinforcement is needed to sufficiently internalize the relevant knowledge and attitudes.

Thirteen types of NGT placement verification methods were identified, with X-ray and the aspirate pH test showing the highest verification accuracy (Jiang et al., 2013). Moreover, the pilot programs showed conducting an aspirate pH test before tube feeding and drug administration to be the most reliable method for verifying NGT placement at the bedside (AACN, 2016; Kunis, 2007; Peter & Gill, 2009). Generally speaking, clinical nurses usually observe gastric aspirates to verify NGT placement. Nurses will insufflate air through the NGT for auscultation if no aspirate is found. However, the whooshing sound in the upper abdomen may originate from the tracheobronchial tree or the pleural cavity (Boeykens et al., 2014). Therefore, the aspirate pH test should be conducted to improve the validity of NGT placement verification.

The effects of the revised standard care procedure on participant attitudes toward NGT placement verification were not significantly better than other outcomes, perhaps because the nurses in the experimental group were required to perform an aspirate pH test to verify NGT placement before each administration or feeding. This additional requirement increases nurse workload, which may explain the rise in attitude scores at the first posttest and then the decline thereafter. With increased proficiency, nurses better understood the importance of adding this procedure in terms of ensuring patient safety, leading to a gradual reduction in related complaints.

Several challenges complicate the process of transferring a synthesis of the evidence into clinical application. These include the additional work stress caused by changing care procedures and inadequate support and recognition from supervisors for the implementation efforts of nurses.

This study is affected by three limitations. First, this research was restricted to the ICUs of one teaching hospital in central Taiwan. Thus, the results may not be generalizable to all critical care nurses. In addition, to avoid cross-contamination, a randomized cluster sampling approach was adopted, which may potentially affect the homogeneity of participants. Finally, the effects of the intervention were evaluated at 1, 2, and 3 months after its conclusion. Thus, the longer-term effects of the intervention remain uncertain.

### Conclusions

This empirical application of research may encourage practitioners to reexamine and reflect on current NGT care practices. The results of this study highlight the feasibility of applying the aspirate pH test in ICU settings. However, this test may not be applicable in all ICUs.

The recommendations for future related research include increasing the sample size and recruiting participants from different hospitals and geographic areas. We suggest that future researchers consider a crossover or self-comparison design to eliminate preexisting differences between two groups. To assess the longer-term effects of the intervention, the second and third posttest times should be extended to 6 and 12 months, respectively. More research should be published on this issue to promote a sufficiently evidence-based NGT placement verification program.

## References

[bib1] American Association of Critical-Care Nurses. (2016). AACN practice alert: Initial and ongoing verification of feeding tube placement in adults. *Critical Care Nurse*, 36(2), e8–e13. 10.4037/ccn2016141 27037348

[bib2] Boeykens K. Steeman E. & Duysburgh I. (2014). Reliability of pH measurement and the auscultatory method to confirm the position of a nasogastric tube. *International Journal of Nursing Studies*, 51(11), 1427–1433. 10.1016/j.ijnurstu.2014.03.004 24731474

[bib3] Bourgault A. M. Heath J. Hooper V. Sole M. L. Waller J. L. & Nesmith E. G. (2014). Factors influencing critical care nurses' adoption of the AACN practice alert on verification of feeding tube placement. *American Journal of Critical Care*, 23(2), 134–143. 10.4037/ajcc2014558 24585162

[bib4] Chan E. Y. Ng I. H. Tan S. L. Jabin K. Lee L. N. & Ang C. C. (2012). Nasogastric feeding practices: A survey using clinical scenarios. *International Journal of Nursing Studies*, 49(3), 310–319. 10.1016/j.ijnurstu.2011.09.014 21974794

[bib5] Cohen J. (1992). A power primer. *Psychological Bulletin*, 112(1), 155–159. 19565683 10.1037//0033-2909.112.1.155

[bib6] DiBardino D. M. & Wunderink R. G. (2015). Aspiration pneumonia: A review of modern trend. *Journal of Critical Care*, 30(1), 40–48. 10.1016/j.jcrc.2014.07.011 25129577

[bib7] Eveleigh M. Law R. Pullyblank A. & Bennett J. (2011). Nasogastric feeding tube placement: Changing culture. *Nursing Time*, 107(41), 14–16. 23251981

[bib8] Fan E. M. Tan S. B. & Ang S. Y. (2017). Nasogastric tube placement confirmation: Where we are and where we should be heading. *Proceedings of Singapore Healthcare*, 26(3), 189–195. 10.1177/2010105817705141

[bib9] Faul F. Erdfelder E. Lang A. G. & Buchner A. (2007). G* power 3: A flexible statistical power analysis program for the social, behavioral, and biomedical sciences. *Behavior Research Methods*, 39(2), 175–191. 10.3758/BF03193146 17695343

[bib10] French S. D. Green S. E. O’Connor D. A. McKenzie J. E. Francis J. J. Michie S. … Grimshaw J. M. (2012). Developing theory-informed behaviour change interventions to implement evidence into practice: A systematic approach using the theoretical domains framework. *Implementation Science*, 7, 38–40. 10.1186/1748-5908-7-38 22531013 PMC3443064

[bib11] Jiang Y. X. Lin F. Y. Kao M. C. Lin R. A. & Wu S. J. (2013). A systematic review of methods for detecting nasogastric tube misplacement after insertion. *The Journal of Long-Term Care*, 17(2), 105–124. (Original work published in Chinese).

[bib12] Ke S. C. Lin F. Y. Hsieh Y. H. Hwu Y. J. & Chang C. N. (2014). Project to increase the nasogastric tube verification practice. *Show Chwan Medical Journal*, 13(3–4), 63–72. 10.3966/156104972014121303002 (Original work published in Chinese)

[bib13] Kunis K. (2007). Confirmation of nasogastric tube placement. *American Journal of Critical Care*, 16(1), 19. 17192522

[bib14] Lin Y. L. Liao C. C. Yu W. P. Chu T. L. & Ho L. H. (2018). A multidisciplinary program reduces over 24 hours of physical restraint in neurological intensive care unit. *The Journal of Nursing Research*, 26(4), 288–296. 10.1097/jnr.0000000000000251 29389807

[bib15] Metheny N. A. & Titler M. G. (2010). Assessing placement of feeding tubes. *The American Journal of Nursing*, 101(5), 36–45. 10.1097/00000446-200105000-00017 11355494

[bib16] Peter S. & Gill F. (2009). Development of a clinical practice guideline for testing nasogastric tube placement. *Journal for Specialists in Pediatric Nursing*, 14(1), 3–11. 10.1111/j.1744-6155.2008.00161.x 19161570

[bib17] Schunk D. H. (2007). *Learning theories: An educational perspective* (5th ed). Upper Saddle River, NJ: Prentice Hall.

[bib18] Stepter C. R. (2012). Maintaining placement of temporary enteral feeding tubes in adults: A critical appraisal of the evidence. *Medsurg Nursing*, 21(2), 61–68. 22666997

[bib19] Stewart M. L. Biddle M. & Thomas T. (2017). Evaluation of current feeding practices in the critically ill: A retrospective chart review. *Intensive and Critical Care Nursing*, 38, 24–30. 10.1016/j.iccn.2016.05.004 27395368

[bib20] Tan L. Chang L. & Wu H. P. (2012). Measuring the pH of gastric aspirate to determine nasogastric tube placement: A systematic review. *Chinese Journal of Practical Nursing*, 31, 57–59. (Original work published in Chinese)

[bib21] Tu C. T. (2017). The basic concept of analysis of covariance. In Tu C. T. (Ed.), *Experimental research methods and analysis of covariance* (1st ed, pp. 32–85). Taipei City, Taiwan, ROC: Wu-Nan Book (Original work published in Chinese)

[bib22] Wu S. J. Lin F. Y. Hwu Y. J. Ke S. C. & Chang C. N. (2016). Evidence utilization of NG tube care in the intensive care units. *Resuscitation & Intensive Care Medicine*, 1(3), 130–141. (Original work published in Chinese)

[bib23] Yang F. H. Lin F. Y. & Hwu Y. J. (2017). Knowledge, attitude, and behavior of Taiwan nurses toward nasogastric tube placement verification. *Cheng Ching Medical Journal*, 13(1), 55–63. (Original work published in Chinese)

